# Global Evolution of Pathogenic Bacteria With Extensive Use of Fluoroquinolone Agents

**DOI:** 10.3389/fmicb.2020.00271

**Published:** 2020-02-25

**Authors:** Miklos Fuzi, Jesus Rodriguez Baño, Akos Toth

**Affiliations:** ^1^Institute of Medical Microbiology, Semmelweis University, Budapest, Hungary; ^2^Unit of Infectious Diseases, Clinical Microbiology and Preventive Medicine, Department of Medicine, Hospital Universitario Virgen Macarena, University of Seville – Biomedicine Institute of Seville (IBiS), Seville, Spain; ^3^Department of Bacteriology, Mycology and Parasitology, National Public Health Center, Budapest, Hungary

**Keywords:** multidrug-resistant pathogens, clonal dynamics, fluoroquinolone resistance, fitness cost, virulence

## Abstract

It is well-established that the spread of many multidrug-resistant (MDR) bacteria is predominantly clonal. Interestingly the international clones/sequence types (STs) of most pathogens emerged and disseminated during the last three decades. Strong experimental evidence from multiple laboratories indicate that diverse fitness cost associated with high-level resistance to fluoroquinolones contributed to the selection and promotion of the international clones/STs of hospital-associated methicillin-resistant *Staphylococcus aureus* (HA-MRSA), extended-spectrum β-lactamase-(ESBL)-producing *Klebsiella pneumoniae*, ESBL-producing *Escherichia coli* and *Clostridioides difficile*. The overwhelming part of the literature investigating the epidemiology of the pathogens as a function of fluoroquinolone use remain in concordence with these findings. Moreover, recent *in vitro* data clearly show the potential of fluoroquinolone exposure to shape the clonal evolution of *Salmonella* Enteritidis. The success of the international clones/STs in all these species was linked to the strains’ unique ability to evolve multiple energetically beneficial gyrase and topoisomerase IV mutations conferring high-level resistance to fluorquinolones and concomittantly permitting the acquisition of an extra resistance gene load without evoking appreciable fitness cost. Furthermore, by analyzing the clonality of multiple species, the review highlights, that in environments under high antibiotic exposure virulence factors play only a subsidiary role in the clonal dynamics of bacteria relative to multidrug-resistance coupled with favorable fitness (greater speed of replication). Though other groups of antibiotics should also be involved in selecting clones of bacterial pathogens the role of fluoroquinolones due to their peculiar fitness effect remains unique. It is suggested that probably no bacteria remain immune to the influence of fluoroquinolones in shaping their evolutionary dynamics. Consequently a more judicious use of fluoroquinolones, attuned to the proportion of international clone/ST isolates among local pathogens, would not only decrease resistance rates against this group of antibiotics but should also ameliorate the overall antibiotic resistance landscape.

## Introduction

We have been witnessing a genuine metamorphosis in the clonal spectra of many multidrug-resistant (MDR) pathogens during the last three decades. While polyclonal situations also occur a few highly successful international clones/sequence types (STs) of various MDR pathogens achieved dominance in multiple geographic regions replacing local strains and posing a serious challenge for the treatment of patients. The advent of the nowadays principal MDR clones commenced with the widespread dissemination of the first international ST of hospital-associated methicillin-resistant *Staphylococcus aureus* (HA-MRSA) the New York-Japan clone (ST5) in the 1990s (Oliveira et al., 2001) which was followed by the emergence of the ribotype 027 clone of *Clostridioides difficile* (Kuijper et al., 2006), the international STs of vancomycin-resistant *Enterococcus faecium* (VRE) (Willems et al., 2005), and the major STs/lineages of ESBL-producing *Klebsiella pneumoniae* (Damjanova et al., 2008) and ESBL-producing *Escherichia coli* (Nicolas-Chanoine et al., 2008). Subsequently we had to face the global clones of MDR *Acinetobacter baumannii* (Zarrilli et al., 2013) and most recently the international STs of MDR *Enterobacter cloacae* (Gomez-Simmonds et al., 2018).

The question arises what could have triggered the metamorphosis of the clonal landscape of MDR pathogens during this time period that resulted in the dominance of a few recently disseminated global “superbugs”?

Virulence factors have been rightly suspected by many authors to have contributed to the emergence of the MDR clones/STs in various pathogens. However, with the exception of the ribotype 027 clone of *C. difficile* in no the other species showed MDR major clone/ST strains greater virulence relative to minor clone isolates and this observation argues strongly against a virulence-based dissemination. Martínez and Baquero (2002) emphasized the primacy of antibiotic resistance over virulence in the selection of international clones in their classic 2002 paper: “If antibiotics are almost ubiquitously present in the hosts, as in an intensive care unit … the spread of the resistant bacteria is favored” while, “.the most virulent bacteria are perhaps less exposed to antibiotics than non-virulent ones” which may account for the lower virulence score of major clone strains in the healthcare setting.

Varying fitness effects (diverse speed of replication^1^) associated with high-level resistance to fluoroquinolones was demonstrated to confer an indirect growth advantage onto the international clone/ST strains of HA-MRSA (Horváth et al., 2012; Knight et al., 2012; Holden et al., 2013), ESBL-producing *K. pneumoniae* (Toth et al., 2014), ESBL-producing *E. coli* (Johnson et al., 2015a, b) and *C. difficile* (Wasels et al., 2015; Vernon et al., 2019) compared with minor clone isolates that should have contributed to the selection of the international clones/STs in these species [reviewed by Fuzi (2016) and Fuzi et al. (2017)]. A “fluoroquinolone connection” could also account for the puzzling time period – the last three decades – for the emergence and dissemination of the international clones and STs. Fluoroquinolones were introduced in many countries in the late 1990s and consumption increased considerably in the early 2000s^2^. We are aware that the emergence and successful spread of resistant clones is probably a multifactorial phenomenon; however, we hypothesize that fluoroquinolones may have played a critical role.

Though all of the international clones/STs had existed prior to the fluoroquinolone era they constituted smaller groups of various pathogens. Otherwise we would have been aware of their extensive dissemination.

Strains of the two largest international clones of HA-MRSA – ST5 and ST22 – were uncommon in both hospitals and the community before the widespread use of fluoroquinolones and their incidence remained low in the community where it is rarely promoted by exposure to these antibiotics (Oliveira et al., 2002; Vandenesch et al., 2003; Wijaya et al., 2006).

Though no reliable data are available on the incidence of the international STs of MDR *K. pneumoniae* in “pre-fluoroquinolone times” strains from the ST131 clone of *E. coli* are well-known to have been isolated from the 1960s across Europe and the United States (Olesen et al., 2014). The available information suggest that the clone’s incidence must have been much lower then compared with its global presence subsequent to the advent of fluoroquinolones (Nicolas-Chanoine et al., 2008; Olesen et al., 2014; Peirano et al., 2014).

Moreover, it is well-established that *C. difficile* ribotype 027 used to be an uncommon clone in North America prior to the beginning of the “fluoroquinolone era” (24). It “was first identified in 1987” as “a minor strain accounting for less than 5% of isolates on surveys, without apparent association with large outbreaks or increased lethality” (McDonald et al., 2005; Louie, 2005).

Though the international MDR clones/STs showed distinct expansions the different pace of evolution and diverse transmission features of individual pathogens might have accounted for the varying onset and time scale of dissemination.

Growth tests, epidemiological observations and genetic data all support a role for fluoroquinolones in the selection and dissemination of the internatinal clones/STs of HA-MRSA, MDR *K. pneumoniae*, MDR *E. coli* and *C. difficile* [reviewed by Fuzi (2016) and Fuzi et al. (2017)]. This paper will present additional information, published most recently on the fitness, epidemiology and genetics of these pathogens and will analyze the role of virulence factors in the spread of these agents. It will be shown that virulence factors play only a subsidiary role in the transmission of MDR bacteria compared with speed of replication in a “high fluoroquinolone exposition.”

### HA-MRSA

It was reported by our group in 2012 and a couple of months later by others that the clonal shifts observed among competing STs of HA-MRSA was in concordance with diverse fitness cost (resulting in diverse speed of replication) associated with high-level resistance to fluoroquinolones (Horváth et al., 2012; Knight et al., 2012; Holden et al., 2013). STs suffering considerable fitness cost upon developing high-level resistance to fluoroquinolones will be replaced by STs retaining much fitness even at high MIC values in “high fluoroquinolone-consumption areas”. In line with experimental results long dominant HA-MRSA clones, especially ST30, ST8, and ST239, have been losing ground to or have been completely replaced by CC5, ST228, and ST22 strains in adult hospital wards across various continents (Mato et al., 2004; Pérez-Roth et al., 2004; Velazquez-Meza et al., 2004; Ma et al., 2006; Amorim et al., 2007; Conceição et al., 2007; Aires-de-Sousa et al., 2008; Knight et al., 2012; Espadinha et al., 2013; Lim et al., 2013; Coombs et al., 2014; Abdulgader et al., 2015; Hsu et al., 2015; Lawes et al., 2015; Zarfel et al., 2016; Chamon et al., 2017; Nikolaras et al., 2018).

Moreover, even the fittest STs are not equal and subtle differences between their speed of replication were also reflected in clonal dynamics. The ST228 clone – that was shown to command inferior fitness associated with resistance to fluoroquinolones relative to the ST5 and ST22 clones (Horváth et al., 2012) – was replaced by ST22 strains in German, Italian, Hungarian and Austrian facilities (Albrecht et al., 2011; Baldan et al., 2012; Grundmann et al., 2014; Zarfel et al., 2016).

The observed “fitness influence” may also account for the characteristic community affiliation of community-associated (CA)-MRSA strains. The substantial fluoroquinolone-associated fitness cost demonstrated with various CA-MRSA STs (Horváth et al., 2012) should prevent the widespread dissemination of CA-MRSA in hospital wards where fluoroquinolones are in extensive use. Consequently most CA-MRSA strains will remain in the community where they can survive and disseminate without having to develop high-level resistance to fluoroquinolones.

Interestingly fitness cost associated with high-level resistance to fluoroquinolones is not static. Some strains from the ST8 clone showed remarkable resilience and proved capable of evolving into a novel major HA-MRSA lineage in the United States (Challagundla et al., 2018a).

The ST8 clone (USA300) was previously considered CA-MRSA, however, a new lineage of the pathogen has recently emerged replacing strains from other STs in the healthcare setting (Challagundla et al., 2018a). The reason for the success of the new ST8 lineage is linked to its skillful genetic evolution. All of the major international STs of MRSA were shown to carry two typical quinolone-resistance determining regions (QRDR) mutations affecting the *gyrA* Ser84 and *grlA* Ser80 residues. These double-serine alterations are characteristic features of both highly successful international HA-MRSA groups of CC5 and ST22 (Holden et al., 2013; Challagundla et al., 2018a).

The replacement of the double-serine residues in the DNA gyrase and topoisomerase IV enzymes involved in the binding of fluoroquinolones is crucial for the preservation of considerable fitness at high MIC values to fluoroquinolones. It was demonstrated in various species that the *gyrA* Ser84 and *grlA* Ser80 (or corresponding) residues are not optimal for the function of the enzymes and their replacement by some other amino acids is associated with a modest fitness gain in the isolates, in a clone-dependent fashion [reviewed by Fuzi et al. (2017)].

The question arises if the double-serine residues are not optimal for the function of gyrase and topoisomerase IV why have these residues been evolutionarily preserved?

Japanese scientists demonstrated in *S. aureus* that these serine residues (in codon 84 in gyrase and codon 80 in topoisomerase IV) confer protection against antibacterial substances of herbal and *Streptomyces* origin (Hiramatsu et al., 2012; Morimoto et al., 2015) permitting survival in diverse environments.

Similarly to the ST5 and ST22 clones the success of the novel lineage of the ST8 strains should be related to their ability to evolve the double-serine QRDR mutations. While the early CA-MRSA ST8 (USA300) strains were either susceptible to fluoroquinolones or carried just one of the serine mutations (*grlA* Ser80) the novel strains harbored also a *gyrA* Ser84 change in a similar fashion to the ST5 and ST22 clones (Holden et al., 2013; Alam et al., 2015; Challagundla et al., 2018b). In their recent review of the USA300 MRSA ST8 clone Challagundla et al. (2018a) also emphasized the relevance of the evolution of the second – *gyrA* Ser84Leu – mutation in the success of the lineage in the United States. Moreover, ST8 strains were reported having evolved double-serine QRDR mutations also in other geographical regions (Khokhlova et al., 2015; Glaser et al., 2016; Wan et al., 2016) that might have contributed to the dissemination of ST8 strains in France (Glaser et al., 2016) and Russia (Gostev et al., 2017). Unfortunately information on the prevalence of QRDR mutations in ST8 MRSA strains are missing from many countries. Data available from some European countries show that ST8 CA-MRSA strains – like other CA-MRSA isolates – are either void of QRDR mutations or carry just a single serine QRDR mutation (Horváth et al., 2012; Lepuschitz et al., 2018).

Though information remains scarce it has to be noted that in some geographical regions some strains of emerging ST239 and ST398 MRSA sublineages also proved capable of developing the double-serine QRDR mutations that may account for the sustenance of these groups in the healthcare setting in some areas (Takano et al., 2008; Lozano et al., 2012; Chakrakodi et al., 2014; Khokhlova et al., 2015).

The superior fitness (speed of replication) of the major international clone strains of HA-MRSA relative to minor clone isolates has serious practical implications. Since these strains multiply faster than isolates from other clones they are expected to achieve higher rates upon occupying novel niches and replacing the local pathogens. This is really what we have been witnessing for more than two decades. The replacement of minor clone strains of HA-MRSA by faster replicating international ST strains showing higher growth rates is well-documented to trigger a significant rise in the incidence of the pathogen (O’Neill et al., 2001; Horváth et al., 2012; Holden et al., 2013; Hsu et al., 2015; Zarfel et al., 2016).

Horváth et al. (2012) and Knight et al. (2012) have demonstrated with *in vitro* propagation assays that the growth rate of international HA-MRSA clone isolates is higher than that of minor ST strains. Since in many countries a large proportion of the HA-MRSA strains belong to the fluoroquinolone-selected major STs a decrease in the use of fluoroquinolones should result in a decline of these major clone strains yielding lower incidences. The overwhelming part of the literature published until 2016 investigating a link between the incidence of HA-MRSA and fluoroquinolone consumption support the existence of an association. The previous literature on the area was reviewed by one of us (Fuzi, 2016). Moreover, a recent report analyzing the impact of antibiotic consumption on the prevalence of MRSA in seven European countries found that the use of both cephalosporins and fluoroquinolones were significantly associated with the occurrence of the pathogen (Kinoshita et al., 2017). This study was performed by using data available in ESAC-NET the European Antimicrobial Consumption database where many countries did not disclose separate figures for antibiotic consumption in the community and in the hospital sector^3^. Nevertheless, it is obvious that a dramatic decline in the rate for MRSA occurred in the United Kingdom. Though we do not have antibiotic consumption data in the hospital sector for the whole country Dingle et al. (2017) published the figures for England where the use of fluoroquinolones in the hospital sector was reduced by close to 50% between 2005 and 2012 (Dingle et al., 2017). During the same period the proportion of MRSA among invasive infections caused by *S. aureus* decreased from 43.6% to 14.0% in the United Kingdom^4^. The reduction must have been partly associated with the more judicious use of fluoroquinolones. Finally Conlon-Bingham et al. (2019) recently observed a link between the use of some antibiotics including fluoroquinolones and the incidence of HA-MRSA in a local hospital-based study. A significant temporal association was demonstrated between HA-MRSA incidence and fluoroquinolone use (coefficient 0.004, *p* < 0.01), with a 3-month lag time (Conlon-Bingham et al., 2019).

We believe that not just fitness cost associated with high-level resistance to fluoroquinolones but fitness in general governs the clonal dynamics of HA-MRSA. Though not all clonal replacements involving MRSA are associated with resistance to fluoroquinolones, fitness costs – in at least some of these clonal shifts – seem also have been involved. During two recent clonal shifts reported in MRSA the fitness of the isolates was investigated and scientists found that strains from the dominant ST/lineage commanded superior replication capacity compared with the replaced isolates (Shang et al., 2016; Li et al., 2018). The latter clonal shift occurred at the expense of mostly fluoroquinolone resistant ST239 isolates which were replaced by faster growing primarily fluroquinolone susceptible ST59 strains. A shift of that nature – obviously – must have taken place in wards where fluoroquinolones were not in extensive use (Li et al., 2018).

In summary, substantial circumstantial evidence suggest that fitness (replication capacity) plays a major – probably crucial – role in the clonal dynamics of MRSA in the healthcare setting in adult facilities. However, the contribution of pathogenicity to the epidemiology of MRSA is rightly considered relevant and therefore we must evaluate the significance of virulence factors relative to replication in the clonal dynamics of the pathogen.

### The Virulence Component in MRSA

Strains from successful international clones/STs of HA-MRSA are in general less virulent than isolates of CA-MRSA which are conversely more susceptible to antibiotics.

Monecke et al. (2011) investigating the virulence factors of a huge number of MRSA strains from a variety of clones/STs clearly showed that the production of the Panton-Valentine leukocidin (PVL) remains a hallmark of CA-MRSA isolates. The production of PVL is not rare among well-known CA-MRSA clones like ST30, ST59, and ST80. However, it will be produced mostly by the fluoroquinolone susceptible CA-MRSA variants of the biggest international HA-MRSA clones/STs of CC5, ST8, and ST22 associated almost exclusively with the small-size *sccmec-*IV/V elements (Rossney et al., 2007; Pinto et al., 2013; Udo et al., 2016; Yang et al., 2019). These observations, the clear distinction in the production of PVL between susceptible CA-MRSA and major clone/ST HA-MRSA strains, strongly suggests that the fitness gain associated with the “non-production of PVL” may assist the usually MDR HA-MRSA strains to disseminate in the healthcare setting.

The “arginine catabolic mobile element” (ACME) enhancing survival of *S. aureus* on the skin of patients was correctly linked to the success of the ST8 (USA300) CA-MRSA clone in the United States (Strauß et al., 2017; Challagundla et al., 2018a). However, interestingly, ACME was missing from strains of the novel lineages of the ST8 HA-MRSA clone some of which have extensively disseminated in the healthcare setting on various continents [reviewed by Strauß et al. (2017)] In addition, ACME remains uncommon in the international clone/ST strains of ST5 and ST22 (Monecke et al., 2011). Moreover, Hsu et al. (2015) recently observed a clonal displacement of the ST239 MRSA clone by ST22 strains in Singapore and a thorough review of the genetic makeup of both clones showed that in contrast to the ST22 isolates many of the ST239 strains harbored the genes of ACME. Thus, greater speed of replication associated with high-level resistance to fluoroquinolones – permitted by the acquisition of the double-serine QRDR mutations – proved more important for clonal superiority than the carriage of ACME.

Nevertheless, the carriage of ACME should certainly confer an advantage to antibiotic susceptible CA-MRSA strains facilitating transmission in a fluoroquinolone-free environment (Aung et al., 2017; Strauß et al., 2017; Murai et al., 2019).

Biofilm is also rightly considered a relevant determinant of clonal dynamics. However, observations published in the related literature show it plays only a secondary role to fitness, i.e., speed of replication.

Though the production of biofilm was suggested to promote the dissemination of the ST22 (EMRSA-15) clone the general biofilm-producing capacity of the ST22 isolates remain inferior compared with those of ST228 and ST8 strains (Baldan et al., 2012) which they readily replace in the healthcare setting (see above).

Biofilm production was observed to advance the dissemination of ST59 MRSA in the community and also in the healthcare setting in pediatric wards which are supposed to be “fluoroquinolone-free” (Yang et al., 2017). These ST59 strains proved strong producers of biofilm, however, more than 80% of the isolates were susceptible to fluoroquinolones and even the resistant strains might have had relatively low MIC values since all of them were isolated from young children (Yang et al., 2017). Nevertheless, despite the production of substantial biofilm these susceptible ST59 strains have not been observed to disseminate in adult wards where fluoroquinolones are supposed to be in use. Moreover, the production of biofilm has also been shown to be influenced by not just the type of the pathogen but also by resistance to macrolides (Sun et al., 2018), previous exposure to antibiotics and some features of the infected patient (Luther et al., 2018).

Baldan et al. (2015) suggested that ST22 HA-MRSA could replace ST228 strains because of a higher rate of hemolysin production. However, they did not consider the “fluoroquinolone” effect. Figure 1 in their paper (Baldan et al., 2015) clearly shows that the incidence of the ST22 strains was in complete agreement with the consumption of fluoroquinolones and it had been previously demonstrated that ST22 strains of HA-MRSA suffer smaller fitness cost upon developing high-level resistance to fluoroquinolones than ST228 isolates (Horváth et al., 2012).

The primacy of fitness (speed of replication) over virulence in MRSA is also reflected by the observation that the acquisition of methicillin resistance by HA-MRSA is associated with an overall downregulation of virulence gene expression linked to a repression of the activity of the accessory gene regulator system (agr) [reviewed by McCarthy et al. (2015)]. The product of the *psm-mec* gene carried by most strains of HA-MRSA on the SCCmec II and III cassettes is responsible for the attenuation of virulence (Kaito et al., 2013).

It is tempting to speculate that the favorable fitness effect gained from the double-serine QRDR mutations combined with the *psm-mec*-induced suppression of multiple virulence genes allowed HA-MRSA to acquire/evolve further antibiotic resistance mechanisms without suffering significant fitness cost permitting the pathogen’s survival in “high-antibiotic” areas.

Strains from the fluoroquinolone resistant lineage of the ST8 MRSA clone in France were reported to carry significantly fewer virulence genes than the fluoroquinolone susceptible ST5 and ST80 isolates (Dauwalder et al., 2008). Thus, strains seem to reduce the carriage/expression of virulence genes as a balancing act for the more important preservation of fitness – if they are capable – when having been “weighted down” by antibiotic resistance.

However, they are not always capable of performing a balancing act. The most virulent clone of *S. aureus* is certainly the ST121 clone harboring a large “armament” of pathogenicity factors (Rao et al., 2015). ST121 is a global clone and an important pathogen in the community, nevertheless, its CA-MRSA variant remains rare (51, 74). Furthermore, high-level resistance to fluoroquinolones has not been reported in ST121 *S. aureus* to date and the clone has never been able to extensively disseminate as a HA-MRSA in hospitals. Thus, it seems, supervirulence has not helped the ST121 clone in the healthcare setting and its obvious inability to preserve fitness upon developing multidrug-resistance might have prevented it from becoming a major pathogen in hospitals.

### Multidrug-Resistant *K. pneumoniae*

Subsequent to the clonal shifts with HA-MRSA we witnessed a substantial “clonal rearrangement” of MDR *K. pneumoniae* in Hungary in 2004–2005 (Damjanova et al., 2008). The investigation of clonal fitness associated with resistance to fluoroquinolones yielded similar results to that with HA-MRSA, however, the difference in loss of robustness proved even greater between minor- and international STs of MDR *K. pneumoniae* than between minor-, and major clones of HA-MRSA (Toth et al., 2014).

Moreover, a clear difference was shown in the use of efflux systems between minor-, and international STs of *K. pneumoniae*. While active efflux could be demonstrated in a Phe-Arg-β-naphthylamide inhibition assay in all of the minor ST strains in which resistance to ciprofloxacin was *in vitro* induced, highly fluoroquinolone resistant major ST isolates proved negative in the test (Toth et al., 2014). Since running an active efflux requires an investment of energy (the area was reviewed recently: Fuzi et al., 2017) it must have impacted the fitness of the fluoroquinolone resistant minor ST isolates (Toth et al., 2014).

Furthermore our previous findings with minor- and major ST strains of *K. pneumoniae* strongly suggest that fitness cost associated with resistance to fluoroquinolones contributed to the widespread dissemination of CTX-M type ESBL genes. We found that all of our fluoroquinolone susceptible minor ST isolates which originally carried plasmids with SHV type ESBL genes eliminated these plasmids upon induction of resistance to ciprofloxacin while strains with plasmids harboring CTX-M type ESBL genes were not cleared from either major-, or minor ST fluoroquinolone resistant isolates (Toth et al., 2014). The process permitting the persistance of the *blaCTX-M-15*-carrying plasmids remains unknown.

In addition, the success of the major ST (ST11, ST15, ST147) MDR *K. pneumoniae* strains was shown to be associated with ability to evolve at least two but rather three energetically favorable QRDR mutations. In contrast to major ST strains minor ST isolates either proved unable to evolve any of these genetic changes or developed just a single alteration (Toth et al., 2014) – a measure reminiscent strongly of observations made with HA-MRSA. Consequently the influence of fluoroquinolones might have substantially contributed to the success of the major clones of *K. pneumoniae*.

Furthermore the carriage of multiple QRDR mutations proved also characteristic for ST258 isolates. Bowers et al. (2015) in their comprehensive review of KPC-producing ST258 *K. pneumoniae* observed: “.all CG258 isolates have the fluoroquinolone resistance-conferring mutations in *GyrA* (Ser83 to Ile) and *ParC* (Ser80 to Ile),” which should have contributed to the global dissemination of carbapenem resistance.

Moreover, a most recent paper characterizing strains of the novel international MDR *K. pneumoniae* sequence type, ST101, showed that almost all of the strains carry three “classic” QRDR mutations supporting further the “fluoroquinolone concept” (Roe et al., 2019). In addition, the carriage of the “double-serine” QRDR mutations was reported characteristic also for the widespread clone of ST307 *MDR K. pneumoniae* (Wyres et al., 2019).

### The Virulence Component in MDR *K. pneumoniae*

Similarly to HA-MRSA the potential role of virulence factors vs robust fitness (high speed of replication) in the clonal dynamics of MDR *K. pneumoniae* need to be assessed.

Interestingly fecal carriage of MDR major ST *K. pneumoniae* strains were reported from various continents in both outpatients and the general population with higher prevalence in developing countries (Zhang et al., 2015; Baraniak et al., 2016; Aghamohammad et al., 2018; Li et al., 2019; Büdel et al., 2019; Pan et al., 2019). In addition, most recently MDR major ST strains of *K. pneumoniae* were observed to colonize more frequently the respiratory system than the gastrointestinal tract (Shu et al., 2019). The factors governing the colonization capacity of MDR major ST strains of *K. pneumoniae* in various sites of the human body remain to be determined.

Though some major international ST (ST11, ST14, ST147, ST258) strains of MDR *K. pneumoniae* are called sometimes hypervirulent or “high-risk” an abundance of papers show that these isolates carry significantly fewer virulence factors than strains from the really highly virulent STs, primarily ST23 and ST65 (Lascols et al., 2013; Bialek-Davenet et al., 2014; Liu et al., 2014; Qu et al., 2015; Yan et al., 2015; Paczosa and Mecsas, 2016; Gomez-Simmonds and Uhlemann, 2017; Gu et al., 2018; Lam et al., 2018b; Turton et al., 2018). Interestingly – despite the large outfit of virulence factors – ST23 and ST65 strains remain minor STs relative to the major clones of MDR *K. pneumoniae* in the healthcare setting (Cantón et al., 2012; Chen et al., 2014; Pitout et al., 2015; Gomez-Simmonds and Uhlemann, 2017; Runcharoen et al., 2017). Moreover it is well-established that strains from the most virulent *K. pneumoniae* sequence type ST23 remain less resistant to antibiotics – primarily to fluoroquinolones – than isolates from the MDR major international STs (Cejas et al., 2014; Qu et al., 2015; Cheong et al., 2016; Yan et al., 2016; Chen Y.T. et al., 2017; Ku et al., 2017; Lu et al., 2017; Sturm et al., 2018; Shen et al., 2019). Moreover, similarly to the most virulent MRSA clone, ST121, no high-level resistance to fluoroquinolones has been reported in any ST23 *K. pneumoniae* strain to date.

Some virulence factors were supposed to be associated with the successful dissemination of the major international STs of MDR *K. pneumoniae*. Holt et al. (2015) suggested that the production of yersiniabactin was associated with the success of the major international ST strains. Nevertheless, the penetrance of yersiniabactin genes is far from complete in major ST isolates (Bialek-Davenet et al., 2014; Dong et al., 2018; Gu et al., 2018; Lam et al., 2018b; Turton et al., 2018; Marques et al., 2019). In contrast the carriage of yersiniabactin genes is a hallmark of ST23 strains and some other minor ST isolates (Lam et al., 2018a) which remain far less common than major ST MDR *K. pneumoniae* strains in the healthcare setting (Damjanova et al., 2008; D’Andrea et al., 2013; Lee et al., 2017; Ko, 2019).

Colibactin was correctly presumed to contribute to the dissemination of ST23 *K. pneumoniae* (Chen Y.T. et al., 2017), however, if colibactin were the most important determinant of transmission of pathogenic *K. pneumoniae* then ST23 and ST65 should make the most common groups since the production of colibactin is much more characteristic for these STs than for other clones including the major MDR international groups (Chen Y.T. et al., 2017; Lam et al., 2018a). In addition, Lam et al. (2018a) observed that the colibactin locus was often disrupted in ST258 isolates and suggested: “.selection against costly colibactin production in hospital adapted strains that already benefit from positive selection under antimicrobial exposure.”

Andrade et al. (2014) suggested that biofilm formation contributed to the advancement of ST11 MDR *K. pneumoniae*. However, Zheng et al. (2018) recently showed that ST23 strains of *K. pneumoniae* produce significantly greater quantities of biofilm than ST11 strains. In addition, Diago-Navarro et al. (2014) demonstrated that ST23 strains also generate much more biofilm than isolates from the global MDR group ST258 which is related to the STT11 clone.

Moreover though aerobactin is considered the prime virulence factor in *K. pneumoniae* (Russo et al., 2015) its prevalence can be low in strains of major international STs (Zhan et al., 2017; Liu et al., 2019).

Nevertheless some gram negative bacteria – including certainly also *K. pneumoniae* – are capable of employing an additional “fitness maneuvre” to advance their performance. Interestingly some major ST *K. pneumoniae* strains, even though showing an MDR phenotype, may harbor more virulence genes than some more susceptible isolates. However, several groups who investigated the integron content in the isolates reported that an extra virulence gene load is significantly associated with the carriage of type 1 integrons conferring resistance to antibiotics (Derakhshan et al., 2016; Xu et al., 2017; Zaki, 2019). Though the promoter types of *K. pneumoniae* integrons have not been investigated to date it is well-established that type 1 integrons usually contain weak promoters in *E. coli* (Vinué et al., 2011; Wei et al., 2013). Weak promoters remain dominant also in class 1 integrons in *Proteus* species and were recently demonstrated to confer a favorable fitness effect on the isolates (Xiao et al., 2019). A similarly favorable fitness effect mediated by class 1 integrons remains most probable in *K. pneumoniae*.

Finally, similarly to MRSA, in community-acquired *Klebsiella* infections the more virulent and less resistant strains of “non-major ST” *K. pneumoniae* will dominate, like ST23 and ST65 (Zhao et al., 2016; Chen Y.T. et al., 2017; Garza-Ramos et al., 2018; Shi et al., 2018) in which high-level resistance to fluoroquinolones has not been reported to date.

In summary, the available data strongly suggest that – similarly to HA-MRSA – the contribution of virulence factors was only supplementary compared with high growth rate to the success of the major STs of MDR *K. pneumoniae* in the adult hospital setting.

### Multidrug-Resistant *E. coli*

One year subsequent to our report demonstrating the impact of fluoroquinolones on the clonal dynamics of MDR *K. pneumoniae* a similar mechanism was proposed for the emergence of the then single global clone/lineage of MDR *E. coli*: ST131 H30R (Johnson et al., 2015a, b). MDR ST131 H30R strains were demonstrated to command a fitness advantage when showing high-level resistance to fluoroquinolones relative to *E. coli* strains from other STs (Johnson et al., 2015a). Moreover in a similar fashion to MDR *K. pneumoniae* a lower efflux pump activity was observed in major ST strains of *E. coli* than in minor clone isolates (Johnson et al., 2015a).

Interestingly, unlike the major international STs of HA-MRSA and MDR *K. pneumoniae* which mostly carry just two or three alterations in the gyrase and topoisomerase IV genes strains from the ST131 H30R lineage carry four or often five QRDR mutations (Fuzi et al., 2017). Individual QRDR mutations in *E.coli* strains will just modestly raise the MIC values for fluoroquinolones, thus, the isolate needs to acquire at least four mutations to achieve a resistance level that allows its survival in a fluoroquinolone environment [area reviewed by Fuzi et al. (2017)]. Moreover the strain has to evolve the multiple QRDR mutations so “cleverly” so as not to compromise its fitness (growth rate) too much (Marcusson et al., 2009). That remains a real challenge that could have been met by only two international MDR lineages of MDR *E. coli* so far.

Apart from ST131 H30R one more group, ST1193, seems to be emerging as a global pathogen (Zhao et al., 2015; Johnson et al., 2018; Valenza et al., 2019). MDR ST1193 strains similarly to MDR ST131 H30R isolates carry multiple (mostly four) QRDR mutations (Wu et al., 2017; Valenza et al., 2019) and show high-level resistance to fluoroquinolones (Johnson et al., 2019).

The proportion of the ST131 H30Rx *E. coli* is usually lower and more variable among clinical *E. coli* isolates than that of the major STs/lineages of HA-MRSA, thus, an epidemiological study investigating the impact of fluoroquinolone use on the incidence of ST131 H30Rx is more difficult to evaluate. Nevertheless, recommendations for a reduced consumption of fluoroquinolones in the United Kingdom starting in 2007^3^ similarly to HA-MRSA (see above) yieded spectacular results. ST131 strains had been reported to dominate the *E. coli* landscape in the United Kingdom prior to 2007 (Lau et al., 2008). However, following the intervention a significant decline was observed in the proportion of the ST131 clone relative to some other STs usually susceptible to fluoroquinolones (Day et al., 2016).

Though Kallonen et al. (2017) suggested that the proportion of ST131 strains of *E. coli* remained “stable” among *E. coli* clinical isolates in the United Kingdom between 2004 and 2012 (Figure 2 in their paper) their study design, unfortunately, did not allow for a solid estimate. First, just the “first ten isolates per site” were collected from each participating laboratory and authors themselves admit that the study could include strains from “local epidemics” (Kallonen et al., 2017). More importantly about one third of the isolates were collected in a single facility starting in 2006 (Kallonen et al., 2017). Consequently the study remains inherently biased with respect to the effect of lower fluoroquinolone consumption that commenced in 2007 (Kallonen et al., 2017).

Patients are for obvious reasons considerably more exposed to fluoroquinolones – and some other antibiotics – in the hospital setting than in the community. The incidence of infections caused by MDR ST131 *E. coli* reflects this difference. The proportion of MDR ST131 strains among isolates obtained from infections is significantly higher in the hospital setting than in the community (Banerjee et al., 2013; Goswami et al., 2018).

Though the proportion of infections caused by MDR ST131 *E. coli* is lower in the community than in the hospital sector the fecal carriage of the pathogen is not rare in outpatients and the general population and can last for extensive time periods (Gurnee et al., 2015; Nakane et al., 2016; Ny et al., 2017; Huang et al., 2018; Morales Barroso et al., 2018; Teunis et al., 2018; Birgy et al., 2019; Meijs et al., 2019). The question arises how can MDR ST131 *E. coli* strains persist in the intestinal flora in the community without ostensible antibiotic exposure?

ST131 strains of *E. coli* may wield some metabolic advantage over other groups of *E. coli* (McNally et al., 2019) but probably more important is their relative insensitivity to colicins that may promote colonization of the gastrointestinal tract (Sharp et al., 2019). Moreover, a low-level exposure of the general population to antibiotics and – occasionally even to ST131 *E. coli* – via food and the environment [reviews by: Riaz et al. (2018); Roth et al. (2019), and Rogers et al. (2011)] would deserve a thorough investigation. Fluoroquinolones are well-established to command an extended degradation half-life in moist environments (Felis et al., 2020).

### The Virulence Component in MDR *E. coli*

Though virulence factors beyond doubt make a relevant contribution to the success of *E. coli* STs several lines of circumstantial evidence suggest that their role – similarly to HA-MRSA and MDR *K. pneumoniae* – remains auxiliary compared with fitness (speed of replication).

Mathers et al. (2015) emphasized that though the carriage of an important virulence gene – the H30 type 1 fimbrial adhesin – has been characteristic for ST131 strains for a long time the clone became globally disseminated only subsequent to the development of high-level resistance to fluoroquinolones.

Though Ciesielczuk et al. (2015) reported that ST131 strains of *E. coli* showed a higher level of virulence in a *Galleria mellonella* model than strains from some other STs (ST69, ST73, ST95), Goswami et al. (2018) demonstrated that the ST131 clone harbors a significantly more limited virulence gene load than ST73 and ST95 isolates which proved mostly susceptible to fluoroquinolones. In addition, a comparative genomic analysis demonstrated that strains from the important sequence types, ST405, ST648, and ST38, which were not tested by Ciesielczuk et al. (2015) carried considerably more virulence factors than ST131 isolates (Shaik et al., 2017). However, despite this lower virulence gene load compared with some STs, it is well-established, that ST131 H30R remains the most prevalent group among MDR *E. coli* in most countries. Moreover, similarly to MRSA and MDR K. *pneumoniae*, the proportion of the major ST131 H30 group remains much lower among fluoroquinolone susceptible *E. coli* strains relative to other STs (Hertz et al., 2016; Yamaji et al., 2018) arguing strongly for a dominant role for fluoroquinolone resistance-associated fitness relative to virulence in promoting dissemination.

ST405 strains of *E. coli* command an impressive virulence gene load (Shaik et al., 2017) and were considered promising candidates for global dissemination (Matsumura et al., 2013). However, the ST405 clone has internationally remained a smaller group relative to ST131 H30 probably due to its inability to evolve a sufficient number of favorable mutations in the gyrase and topoisomerase IV genes. While ST131 H30R and ST1193 strains carry four-to-five QRDR mutations ST405 isolates, similarly to the “moderately disseminated” ST410 strains, have evolved just three alterations (Mavroidi et al., 2012; Alouache et al., 2014; Roer et al., 2018) that is not supposed to confer high-level resistance to fluoroquinolones (Marcusson et al., 2009) preventing thereby their extensive spread. Nevertheless, three QRDR mutations may allow a lower level persistance of the clone in a fluoroquinolone environment. Furthermore, some strains of the ST405 group, similarly to ST8 MRSA, may evolve (or might have evolved) an additional favorable QRDR mutation that may turn or (may be turning) the clone into a prime global player. Strains from additional *E. coli* STs may also prove capable of evolving four (or more) favorable QRDR mutations and start to extensively disseminate in fluoroquinolone environments.

Interestingly Cha et al. (2016) observed a greater virulence gene load in ST131 H30 isolates compared with non-ST131 strains of *E. coli*. However, 71.1% of the non-ST131 isolates were also resistant to ciprofloxacin that should have been associated with a considerable fitness cost in these strains preventing the carriage of a substantial virulence gene load.

ST131 H30 strains are assigned to different virotypes determined by their virulence gene profiles (Blanco et al., 2013; Dahbi et al., 2014). Interestingly virotypes B and D associated with a higher virulence gene load are less frequent than virotypes C and A (Merino et al., 2017). Virotype C strains of ST131 H30 Rx – commanding the smallest virulence gene load – are well-established to be the most common worldwide (Blanco et al., 2013; Nicolas-Chanoine et al., 2014; Merino et al., 2017).

Though virotype C strains of ST131 H30 strains remain most frequent a few groups reported a higher incidence for virotype A isolates (Olesen et al., 2014; Ludden et al., 2015; Jamborova et al., 2018). Virotype A strains harbor a somewhat greater virulence gene load compared with virotype C. This extra gene load is supposed to be associated with fitness cost, thus, the question arises how could these isolates exceed equally resistant or less resistant virotype C strains?

The “integron effect” – observed also with MDR *K. pneumoniae* (see above) – may account for the circumstance. Class 1 integrons are common in *E. coli* (Copur-Cicek et al., 2014; Oliveira-Pinto et al., 2017) including ST131 isolates (Pérez-Etayo et al., 2018) and it is well-established that they are mostly associated with weak promoters (Vinué et al., 2011; Wei et al., 2013). It was demonstrated that these “weak-promoter integrons” are “low-cost structures” in *E. coli* and their carriage influences the fitness of the pathogen (Lacotte et al., 2017). The “integron effect” is supported by the high-quality report of Jamborova et al. (2018) who performed a comprehensive investigation of a large number of ST131 H30 isolates and found that virotype A was the most common variant among the highly resistant strains. However, they also tested the integron content of the isolates and unequivocally showed that, similarly to MDR *K. pneumoniae*, in their collection, the more virulent virotype A isolates carried significantly more type 1 integrons than the virotype C strains.

A few reports were published on ST131 strains harboring a greater virulence gene load than isolates from other STs (Johnson et al., 2009; Blanco et al., 2011; Colpan et al., 2013), however, these observations were made with *E. coli* ST131 isolates which were susceptible to several groups of antibiotics that could have permitted the carriage of a greater virulence gene load. In addition, López-Cerero et al. (2014) showed that “the severity of sepsis, bacteraemia and mortality were similar among ST131 and non-ST131” *E. coli* isolates.

Moreover, Johnson et al. (2019) recently reported that the ST1193-H64 major *E. coli* lineage disseminating extensively in the United States carried a more modest virulence gene load compared with other isolates further supporting the view that virulence factors play only an auxiliary role relative to fitness in determining the transmissibility of pathogens in a fluoroquinolone environment.

In summary we believe that in line with observations obtained with HA-MRSA and MDR *K. pneumoniae* fitness cost associated with resistance to fluoroquinolones is involved more prominently in the dissemination of the global STs of MDR *E. coli* than virulence factors. However, the distinction in virulence between hospital-associated and community-acquired strains is not as unequivocal as with MRSA and MDR *K. pneumoniae* because in contrast to these agents CA-*E. coli* strains, as primarily urogenital pathogens, are often exposed to fluoroquinolones and other antibiotics impacting the fitness of the isolates in a clone-dependent fashion. This is the reason why in contrast to MRSA and MDR *K. pneumonaiae* the fluoroquinolone-selected clone of MDR *E. coli*, ST131, may feature prominently in community-acquired infections (Blanco et al., 2013; Doi et al., 2013; Nicolas-Chanoine et al., 2014; Chong et al., 2018; Jamborova et al., 2018; Matsukawa et al., 2019).

### C. difficile

Two groups have demonstrated that ability to evolve a particular, energetically favorable *gyrA* mutation (Thr82Ile) is responsible for the rapid replication of the major international ribotypes of *C. difficile* when showing high-level resistance to fluoroquinolones (Wasels et al., 2015; Vernon et al., 2019). *C. difficile* does not carry topoisomerase IV genes, thus, a single mutation in the gyrase gene proved sufficient to confer both high-level resistance to fluoroquinolones and a favorable fitness effect.

Wasels et al. (2015) introduced the *gyrA* Thr82Ile mutation – typical for all major *C. difficile* ribotypes (Spigaglia et al., 2010) – into a non-major clone strain of *C. difficile* and found that the isolate suffered just a minimal (2–3%) fitness cost upon developing resistance to fluoroquinolones. In contrast some other QRDR mutations in the same isolate were associated with a considerable loss of robustness. Authors correctly predicted that the favorable fitness effect could have facilitated the dissemination of this particular clone of *C. difficile.*

Vernon et al. (2019) compared the fitness (speed of replication) of seven fluoroquinolone resistant ribotype 027 strains carrying the *gyrA* Thre82Ile mutation with that of their fluoroquinolone susceptible parent isolates void of the genetic alteration and experienced a fitness gain of 8–22% across the isolates. This paper attests that the fitness effect associated with a favorable QRDR mutation can be substantial and is both clone-, and strain specific.

The salience of the *gyrA* Thr82Ile mutation is also reflected by a clonal shift that occurred recently in South Korea. Korean scientists demonstrated that clones which are considered minor in Europe and carry an energetically less favorable mutation will become major clones/ribotypes when they are capable of evolving the Thr82Ile *gyrA* mutation and will replace other ribotypes harboring energetically detrimental *gyrA* alterations (Lee et al., 2014). Consequently, the dissemination of a particular clone was governed by the strains’ ability to evolve the favorable QRDR mutation.

Moreover, in the United Kingdom a campaign was launched in 2007 to restrict the use of fluoroquinolones to reduce the incidence of *C. difficile* (Wilcox et al., 2012). The campaign proved highly successful: the incidence of *C. difficile* infections dropped dramatically during the next couple of years (Wilcox et al., 2012; Dingle et al., 2017). Interestingly, the proportion of the major clone strains also declined significantly and polyclonalilty expanded during the same time period demonstrating clearly the association of major clones with the use of fluoroquinolone type antibiotics (Wilcox et al., 2012). A similar decrease in the rates for HA-MRSA and ST131 *E. coli* subsequent to the reduction of fluoroquinolone use (see above) was also observed.

Furthermore Sarma et al. (2015) reported that a significant decrease in the consumption of fluoroquinolones resulted in a partial replacement of strains from some major clones (all of them characteristically carrying the favorable *gyrA* Thre82Ile mutation) by minor clone isolates.

The observation that the incidence of ribotype 027 strains remains significantly lower in pediatric units (where fluoroquinolones are not supposed to be used) compared with adult wards (McFarland et al., 2016) remains an additional argument for the “fluoroquinolone effect” selecting major clones.

Minor clone strains of *C. difficile* are well-known to often remain susceptible to fluoroquinolones (Wiuff et al., 2011; Knight et al., 2015; Seugendo et al., 2018) or evolve various mutations in the *gyrB* gene and display reduced resistance to this group of antibiotics (Lee et al., 2014; Wasels et al., 2015; Shaw et al., 2019; Vernon et al., 2019). Though some of the genetic alterations in *gyrB* will confer some fitness gain onto the isolates (Wasels et al., 2015; Vernon et al., 2019), it is also well-established that these mutations will accord just a low-level resistance to moxifloxacin rendering the isolates “vulnerable” to exposure (Wasels et al., 2015; Vernon et al., 2019).

The recently observed widespread dissemination of the ribotype 017 clone of *C. difficile* in Asia (Imwattana et al., 2019) may also be associated with the “fluoroquinolone influence”. Ribotype 017 and other expanding-clone strains were reported to mostly carry the favorable *gyrA* Thr82Ile mutation in various Asian countries (Lee et al., 2014; Wang et al., 2018).

It was recently suggested that efficient trehalose metabolism might have contributed to the advance of some clones of *C. difficile* (Collins et al., 2019). However, Eyre et al. (2019) demonstrated that many STs of *C. difficile* share similar metabolic pathways and some of these clones remain extremely rare, thus, the contribution of the “trehalose effect” should be slight compared with that of fluoroquinolones.

### The Virulence Component in *C. difficile*

Apart from favorable fitness various virulence factors produced by ribotype 027 strains have certainly contributed to the clone’s dissemination (Stabler et al., 2009; Valiente et al., 2014). Moreover, strains of additional major ribotypes (001 and 106) have also been shown to command superior virulence relative to many other clones (Vohra and Poxton, 2011). Nevertheless, superior virulence could not prevent the demise of strains from any of these major ribotypes once the selecting pressure of fluoroquinolone exposure ceased/diminished (Wilcox et al., 2012; Lee et al., 2014; Sarma et al., 2015; Dingle et al., 2017).

Finally the question arises: if the *gyrA* Thr82Ile mutation will substantially enhance the fitness of ribotype 027 strains (Vernon et al., 2019) how can isolates from this clone be replaced by minor clone strains upon cessation of fluoroquinolone exposure? The answer is that 027 strains replicate far slower – perhaps as a consequence of producing excessive quantities of toxin – than minor clone isolates. This was clearly established by Carlson et al. (2013) conducting propagation assays with strains of *C. difficile* from diverse genetic backgrounds. They found that “isolates of ribotype 027 produced higher levels of toxin and exhibited slower growth compared to other ribotypes”. This may account for the swift replacement of ribotype 027 strains by isolates from minor clones upon decreased use of fluoroquinolones.

Moreover Carlson et al.’s (2013) report clearly shows that toxin production is not related to transmissibility in *C. difficile*. Carlson et al. (2013) found an abundance of strains from diverse genetic backgrounds which, similarly to ribotype 027 strains, produced large quantities of toxin, nevertheless, their prevalence remained very low or were single isolates and no data is available on any of these ribotype strains carrying the favorable *gyrA* Thr82Ile mutation.

Since major ribotype strains feature prominently in *C. difficile* infections it is well-established that a reduction in the use of fluoroquinolones results in a decline in the incidence of the pathogen (area was reviewed by one of us) (Fuzi, 2016).

### Other Species

All bacterial species causing human infections and exposed to the “fluoroquinolone effect” particularly in adult hospital wards have to adapt to the fluoroquinolone environment by evolving resistance mechanisms. We have seen how MRSA, MDR *K. pneumoniae*, MDR *E. coli* and *C. difficile* responded to the challenge: exclusively strains from a few international/global clones/STs proved capable of developing favorable QRDR mutations and emerged commanding superior fitness and replacing minor clone/ST strains in fluoroquinolone environments. The available literature strongly suggest that the major international clones/STs of additional MDR pathogens might also have been shaped by the fluoroquinolone effect. The related literature on MDR *E. faecium* (VRE) was recently reviewed (Fuzi, 2016; Fuzi et al., 2017).

Although *Streptococcus pneumoniae* is primarily a community-acquired pathogen the extensive use of fluoroquinolones might have contributed to the selection of some clones of the species which managed evolved the energetically favorable double-serine mutations. *S. pneumoniae* strains from these clones proved prevalent in some European, Asian and Latin-American countries (Canton et al., 2003; de la Campa et al., 2009; Hsieh et al., 2010; Ardanuy et al., 2014; Chen H.H. et al., 2017), but could not disseminate in others (Ceyssens et al., 2016; Metcalf et al., 2016; Schmitz et al., 2017). The most widespread “fluoroquinolone resistance-influenced” *S. pneumoniae* sequence type is certainly the ST81 group (de la Campa et al., 2009; Hsieh et al., 2010; Chen H.H. et al., 2017).

Moreover, international STs showing high-level resistance to fluoroquinolones were reported also in *Neisseria gonorrhoeae* with three or more QRDR mutations, including consistently the double-serine alterations (Cámara et al., 2012; Chen et al., 2013; Endimiani et al., 2014; Kubanov et al., 2016). These MDR *N. gonorrhoeae* STs are also suspect of having been influenced by diverse fitness cost associated with resistance to fluoroquinolones.

Furthermore, several papers were recently published on fluoroquinolone resistant *Haemophilus influenzae* strains carrying multiple QRDR mutations and showing clonal relatedness (Kuo et al., 2014; Puig et al., 2015; Fuursted et al., 2016) making these groups promising candidates for fluoroqinolone-selected pathogens.

Though enteric pathogens are less exposed to fluoroquinolones a lineage of fluoroquinolone resistant *Salmonella* Kentucky (ST198) might have been selected by fluoroquinolone pressure in the veterinary sphere. Isolates in this international ST also carry multiple QRDR mutations including the double-serine alterations (Le Hello et al., 2013) that should confer a fitness advantage in a fluoroquinolone environment.

In addition American scientists recently investigated *in vitro* the diverse mechanisms and related varying fitness costs associated with resistance to fluoroquinolones in *Salmonella* Enteritidis. Their findings remain in complete agreement with those obtained with MDR *K. pneumoniae* and MDR. *E. coli* (Toth et al., 2014; Johnson et al., 2015a, b). The single isolate acquiring three QRDR mutations – including the double-serine alterations – retained much fitness (speed of replication) and used little efflux. All the other strains evolved either fewer-, or “non-double-serine” QRDR mutations, employed more efflux and suffered significant fitness costs (Vidovic et al., 2019). Consequently a few proportion of *S. enteritidis* isolates are potentially capable of generating major clones if strains of the serotype were extensively exposed to fluoroquinolones.

*Campylobacter jejuni* has also been exposed to the fluoroquinolone effect, however, this pathogen proved more adept relative to other bacteria to comply with the challenge, certainly due to the hyperplasticity of its genome (area was reviewed by us) (Fuzi, 2016; Fuzi et al., 2017). Consequently no major clones of *C. jejuni* are known to have been selected by fluoroquinolones.

In addition, the involvement of fluoroquinolone resistance-associated fitness cost in the selection of the global/international clones of MDR *A. baumannii, Enterobacter cloacae* and MDR *Pseudomonas aeruginosa* would deserve a thorough investigation.

## Discussion

In summary fluoroquinolones have been shown to select for the major international clones/STs of various MDR pathogens and have potentially affected all species which were exposed to these antibiotics for extensive time periods. Since the evolvement of the double-serine QRDR mutations were often associated with a fitness gain – though in a clone-dependent fashion – the assumption of high-level resistance to fluoroquinolones permitted the major clone/ST isolates to acquire resistance mechanisms against additional groups of antibiotics without suffering a substantial loss of vitality. This is in sharp contrast to minor clone/ST isolates which proved less capable of evolving energetically favorable QRDR mutations and – consequently – had to apply alternative mechanisms for developing resistance to fluoroquinolones, like enhanced efflux, that requires considerable energy. This was, however, associated with substantial fitness cost resulting in compromised growth that allowed the major clone/ST isolates to replace minor clone/ST strains in fluoroquinolone environments. The faster growth rate of these newly dominant major clone/ST isolates then brought about a steep rise in the incidence of MDR pathogens.

Moreover, the available data clearly show that fitness cost suffered by minor clone/ST strains can hardly be recovered by compensatory mutations. Toth et al. (2014) failed to reverse lost fitness in fluoroquinolone resistant minor ST strains of *K. pneumoniae*. Furthermore, it should not be an accident that the clonal landscape of multidrug-resistant pathogens remains largely stable in the hospital sector. If minor clone strains could easily reverse the fitness cost associated with resistance to fluoroquinolones by evolving compensatory mutations novel international clones of various multidrug-resistant pathogens hailing from previous minor ST strains should regularly emerge. However, this is what we have not been witnessing. What we see is just the infrequent dissemination of previous minor clone strains which with some delay proved competent to evolve the energetically favorable QRDR mutations (see above).

We believe the data presented in the paper demonstrate the crucial role fluoroquinolones played in the selection of the international clones/STs of MDR bacteria. Nevertheless, a variety of other groups of antibiotics are well-established to have also promoted the dissemination of MDR pathogens including strains of the international clones of HA-MRSA (Monnet et al., 2004; Lawes et al., 2015), MDR *K. pneumoniae* (da Silva et al., 2012; Ryu et al., 2018), MDR *E. coli* (López-Lozano et al., 2019) and *C. difficile* (Owens et al., 2008; Vardakas et al., 2016). However, the impact of these antibiotics remain second rate relative to that of fluoroquinolones as shown above. The comprehensive review of this literature is beyond the scope of this paper.

Interestingly virulence-, and colonization factors seem to have exerted only a smaller impact relative to growth rate on the dissemination of major bacterial clones/STs in adult hospital wards. This observation is not surprising and remains consistent with features of an environment characterized by the extensive use of antibiotics, primarily that of fluoroquinolones. In this “high-antibiotic environment” a favorable fitness (high growth rate) will confer a considerable advantage onto a particular MDR isolate vs. other competing MDR strains from the same species showing superior virulence/colonization efficiency but inferior replication capabilities. Moreover, an MDR isolate with relatively lower virulence but almost “wild-type fitness” will also command considerable advantage against a highly virulent and fit strain that remains susceptible to antibiotics. Though the latter strain should transmit more readily it can be – in contrast to our “super bug” – easily controlled in the hospital setting with antibiotics.

Consequently, it is not an accident that the most widely disseminated types of the major international ST/lineage strains of HA-MRSA, MDR. *K. pneumoniae* and MDR *E. coli* show less virulence than isolates from many other STs/lineages of the same species (see above). *C. difficile* remains an exception as ribotype 027 strains are highly toxic, however, the extensive dissemination of this clone was also the consequence of substantial fitness gain conferred by the *gyrA* Thr82Ile mutation (Vernon et al., 2019) and not the production of excessive toxin.

Nevertheless, the situation is different in some “low-antibiotic areas” in the community. In a “low-antibiotic” sphere pathogens can afford to remain less resistant and consequently retain fitness and harbor more virulence factors. Since in these areas antibiotics will usually not restrict the bacteria the virulence factors should confer a considerable advantage onto the pathogen that is reflected in the well-established high incidence of virulent and “non-major ST” strains (CA-MRSA, *K. pneumoniae*) in community-acquired infections (see above).

Excessive virulence is well-established even in a low-antibiotic environment to be actually detrimental for the evolutionary success because killing the host will arrest further propagation of the infectious agent. So the equilibrium must be maintained to avoid excessive expression or acquiring too lethal combinations of pathogenic traits that might impact the virulence of both community-acquired and healthcare-associated pathogens.

The fluoroquinolone effect implies that in a fluoroquinolone-free hospital environment the incidence of the international clone/ST strains should decrease. This is exactly what has been observed with both HA-MRSA and *C. difficile*, the two MDR pathogens in which major ST strains comprise the highest proportion of the respective species in adult hospital wards. The regress of the major clones of both pathogens was demonstrated to have been associated with a decline in the incidence of both HA-MRSA and *C. difficile* upon a more judicious use of fluoroquinolones (see above).

The impact of a reduction in the consumption of fluoroquinolones on the incidence of major clone pathogens from other species remains less conspicuous since the proportion of major clone/ST bacteria is smaller among these clinical isolates. Nevertheless, some positive effect should also be observed with other species. Further epidemiological studies involving the judicious use of fluoroquinolones and investigating the clonal composition of the local pathogens are needed.

Finally, the judicial use of fluroquinolones will ameliorate resistance rates only if infection control measures are complied with and poor hygiene will not compromise the expected favorable outcome.

## Data Availability Statement

The raw data supporting the conclusions of this article will be made available by the authors, without undue reservation, to any qualified researcher.

## Author Contributions

The manuscript was based on MF’s perception and he did much of the writing. JR and AT contributed significantly to both writing the manuscript and raising its professional standard by making relevant supplements.

## Conflict of Interest

The authors declare that the research was conducted in the absence of any commercial or financial relationships that could be construed as a potential conflict of interest.

## Footnotes

1 The term fitness always refers to speed of replication in the manuscript.

2 https://ecdc.europa.eu/en/about-us/partnerships-and-networks/disease-and-laboratory-networks/esac-net

3 https://ecdc.europa.eu/en/antimicrobial-consumption/surveillance-and-disease-data/database

4 https://ecdc.europa.eu/en/about-us/partnerships-and-networks/disease-and-laboratory-networks/ears-net
